# Supplementary material for: Normalization of the ATP1A1 Signalosome Rescinds Epigenetic Modifications and Induces Cell Autophagy in Hepatocellular Carcinoma
Source: Cells. 2023 Sep 27;12(19):2367. doi: 10.3390/cells12192367 (PMC10572209; doi:10.3390/cells12192367)
Supplement: Supplementary file 1 [file cells-12-02367-s001.zip › cells-2579243-supplementary.pdf]

Supplementary files

Normalization of the ATP1A1 Signalosome Rescinds Epigenetic Modifications and Induces Cell Autophagy in MASH-Related Hepatocellular Carcinoma

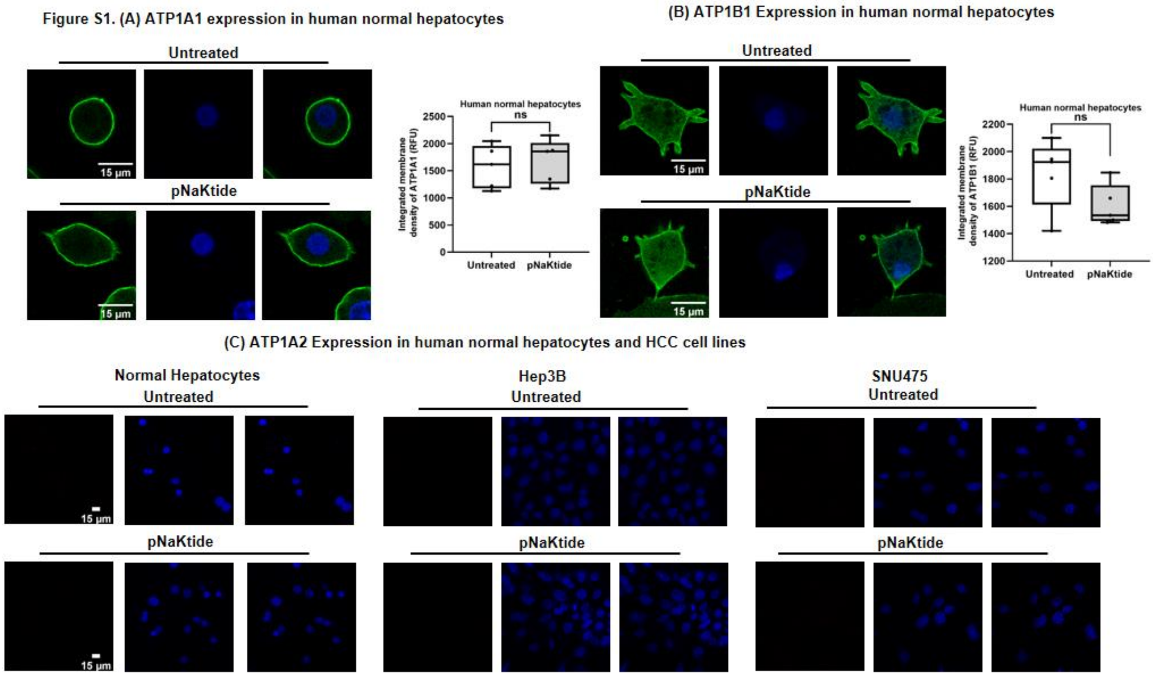

Figure S1. *ATPase subunits expression in human normal hepatocytes and two human HCC cell lines.* No significant differences were observed in ATP1A1 and ATP1B1 subunits of normal hepatocytes treated with pNaKtide as compared to untreated group. In addition, ATP1A2 subunit of both normal hepatocytes and HCC cell lines showed no significant changes under treated or untreated conditions. *A)* Confocal images of ATP1A1 expression in human normal hepatocytes. *B)* Confocal images of ATP1B1 expression in human normal hepatocytes. *C)* Confocal images of ATP1A2 expressions in human normal hepatocytes and human HCC cell lines (Hep3B and SNU475). Results are shown as box-whisker plots (*n*=5, statistical analysis by t-test). Scale bar=15µm.

Figure S2. Nuclear vs Cytoplasmic FoxO1 expression in Human Normal Hepatocytes

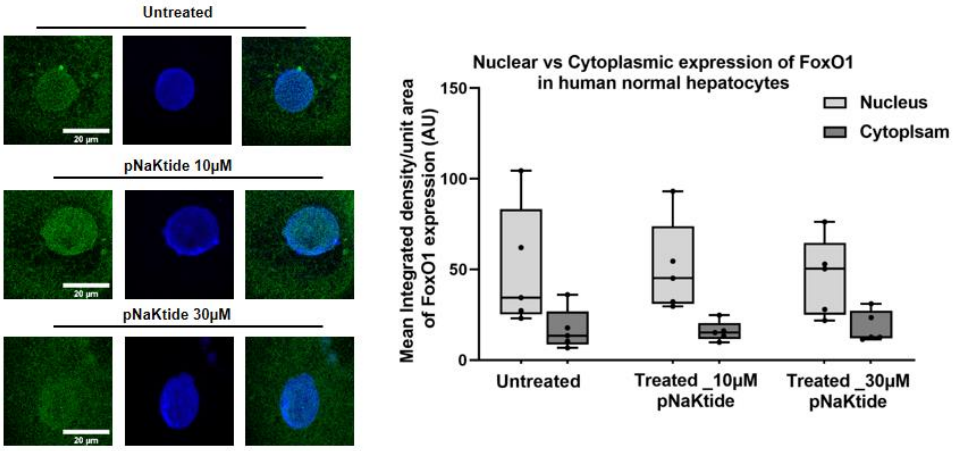

Figure S2. *FoxO1 in human normal hepatocytes.* No significant changes were observed in the nuclear translocation of FoxO1 in the pNaKtide-treated group when compared to untreated human normal hepatocytes. Confocal microscopy images of FoxO1 in human normal hepatocytes (n=5, Results are shown as box-whisker plots. Statistical analysis by ANOVA and Turkey's Post hoc test). Scale bar=20μm.

Table S1. Key reagents and chemicals used in this study. Antibodies against the indicated protein as well as other reagents/chemicals used in this study are as listed below. Their catalogue number, source and the dilutions used in the experiments are as listed.

ANTIBODY/REAGENT/CHEMICAL	Source	Reference	Description	Host Sp.	IHC	ICC/IF	WB
Histone Extraction Kit	Abcam	Catt ab113476	ELISA	N/A	N/A	N/A	N/A
Histone H3 (acetyl K9) Quantification Kit (Colorimetric)	Abcam	Catt ab115104	ELISA	N/A	N/A	N/A	N/A
Histone H3 (tri-methyl K9) Quantification Kit(Colorimetric)	Abcam	Catt ab115064	ELISA	N/A	N/A	N/A	N/A
Anti-Histone H3 (acetyl K9) antibody - ChIP Grade	Abcam	Catt ab10812	Polyclonal	Rabbit	N/A	1:700	
Anti-Histone H3 (tri methyl K9) antibody - ChIP Grade	Abcam	Catt ab8898	Polyclonal	Rabbit	N/A	1:500	N/A
Autophagy ELISA kit (LC3-II Quantitation	MYBIOSOURCE	MBS169564	ELISA	N/A	N/A	N/A	N/A
BioCell Protein Carbonyl Assay Kit	Biocell Corporation	Catt P133	ELISA	N/A	N/A	N/A	N/A
GSH Assay Kit (Colorimetric)	Abcam	Catt ab239727	ELISA	N/A	N/A	N/A	N/A
alpha 1 Na/K-ATPase	Millipore	Catt 05-369	Monoclonal primary	Mouse	N/A	1:100	N/A
Anti-Na+/K+ ATPase β-1 Antibody, clone C464.8	Millipore	Catt 05-382	Monoclonal	Mouse	N/A	N/A	1:1000
FoxO1(C29H4)Rabbit mAb	Cell Signaling Technologies	Catt#2880	Monoclonal	Rabbit	N/A	N/A	1:250
Anti-Alpha 2 Na^{+}/K^{+} ATPase Antibody	Alomone labs	Catt ANP-002	Polyclonal	Rabbit	N/A	1:100	1:1000
Autophagy ELISA Kit (LC3-II Quantitation)	MYBIOSOURC E	Catt MBS169564	ELISA	N/A	N/A	N/A	N/A
Goat Anti-Rabbit IgG H&L (Alexa Fluor® 594)	Abcam	Catt ab150080	Polyclonal Secondary	Goat	1:500	1:100	N/A
Goat Anti-Rabbit IgG H&L (Alexa Fluor® 488), preabsorbed	Abcam	Catt ab150081	Polyclonal Secondary	Goat	N/A	1:500	N/A
Mouse monoclonal antibody anti Na/K-ATPase (α6f)	Developmental Studies- University of Iowa		Monoclonal primary	Mouse	N/A	N/A	1:1000
ATP1B1 Monoclonal Antibody (M17-P5-F11)	Invitrogen	Catt MA3-930	Monoclonal	Mouse	N/A	1:200	N/A
Autophagy Assay Kit	Abcam	Catt ab139484	Fluorescence/Confocal Micoscropy		N/A	N/A	N/A

Table S2. List of major genes dysregulated in Hep3B cell line vs. response to pNaKtide treatment.

Gene_Name	Gene symbol	Adj.p-value	Fold Change	Mean expression Hep3B pNaKtide Treated	Mean expression Hep3B Untreated
**Upregulated genes**					
Activating transcription factor 3	ATF3	8.44E-59	3.487253	3541.213	315.4904
Growth arrest and DNA damage inducible alpha	GADD45A	3.50E-11	1.919932	1103.418	291.8637
Early growth response 1	EGR1	0.001097	2.938658	5863.863	764.8719
Early growth response 2	EGR2	4.90E-07	4.807229	67.71237	2.428744
Mitogen-activated protein kinase 8 interacting protein 1 pseudogene 2	MAPK8IP1P2	0.002549	3.633838	141.9453	11.38391
Sirtuin 6	SIRT6	0.010402	0.651333	542.3259	345.2675
ENTPD3 antisense RNA 1	ENTPD3-AS1	0.007857	1.561042	35.15685	11.83365
Ras related glycolysis inhibitor and calcium channel regulator	RRAD	0.011089	2.488686	24.51084	4.328241
WAP four-disulfide core domain 3	WFDC3	1	4.379217	3.615986	0
Phosphoglycerate mutase family member 4	PGAM4	1	1.732392	11.83512	3.609913
**Downregulated genes**					
**Gene_Name**	**Gene symbol**	**Adj.p-value**	**Fold Change**	**Mean expression Hep3B pNaKtide Treated**	**Mean expression Hep3B Untreated**
High mobility group box 1 pseudogene 14	HMGB1P14	1	-4.68926	0	4.958786
Centrosomal protein 290	CEP290	0.013149	-0.82172	230.2352	407.4144
Zinc finger protein 727	ZNF727	1	-3.317	1.310424	12.65315
Centromere protein F	CENPF	0.014018	-0.62986	4353.742	6737.5
Glypican 6	GPC6	0.015994	-0.60783	1749.369	2666.319
Coiled-coil domain containing 87	CCDC87	1	-1.89191	3.167282	11.65714
WNK lysine deficient protein kinase 1	WNK1	0.032694	-0.74451	5697.554	9545.618
Ubiquitin specific peptidase 8	USP8	0.048214	-0.43553	894.1294	1210.036
Cytidine/uridine monophosphate kinase 2	CMPK2	1	-4.18654	0.175243	5.230682
Kinetochore associated 1	KNTC1	0.063337	-0.47438	1430.311	1986.996
